# Occupancy of Norepinephrine Transporter by Duloxetine in Human Brains Measured by Positron Emission Tomography with (*S,S*)-[^18^F]FMeNER-D_2_

**DOI:** 10.1093/ijnp/pyx069

**Published:** 2017-08-03

**Authors:** Sho Moriguchi, Harumasa Takano, Yasuyuki Kimura, Tomohisa Nagashima, Keisuke Takahata, Manabu Kubota, Soichiro Kitamura, Tatsuya Ishii, Masanori Ichise, Ming-Rong Zhang, Hitoshi Shimada, Masaru Mimura, Jeffrey H Meyer, Makoto Higuchi, Tetsuya Suhara

**Affiliations:** Department of Functional Brain Imaging Research, National Institute of Radiological Sciences, National Institutes for Quantum and Radiological Science and Technology, Chiba, Japan (Drs Moriguchi, Takano, Kimura, Nagashima, Takahata, Kubota, Kitamura, Ishii, Ichise, Zhang, Shimada, Higuchi, and Suhara); Department of Neuropsychiatry, Keio University School of Medicine, Tokyo, Japan (Drs Moriguchi, Takahata, and Mimura); Research Imaging Centre, Centre for Addiction and Mental Health, Toronto, Canada (Drs Moriguchi and Meyer); Department of Psychiatry, National Center of Neurology and Psychiatry, Tokyo, Japan (Dr Takano); Department of Clinical and Experimental Neuroimaging, Center for Development of Advanced Medicine for Dementia, National Center for Geriatrics and Gerontology, Obu, Japan (Dr Kimura)

**Keywords:** PET, *(S,S)*-[^18^F]FMeNER-D_2_, duloxetine, occupancy, norepinephrine transporter

## Abstract

**Background:**

The norepinephrine transporter in the brain has been targeted in the treatment of psychiatric disorders. Duloxetine is a serotonin and norepinephrine reuptake inhibitor that has been widely used for the treatment of depression. However, the relationship between dose and plasma concentration of duloxetine and norepinephrine transporter occupancy in the human brain has not been determined. In this study, we examined norepinephrine transporter occupancy by different doses of duloxetine.

**Methods:**

We calculated norepinephrine transporter occupancies from 2 positron emission tomography scans using (*S,S*)-[^18^F]FMeNER-D_2_ before and after a single oral dose of duloxetine (20 mg, n = 3; 40 mg, n = 3; 60 mg, n =2). Positron emission tomography scans were performed from 120 to 180 minutes after an i.v. bolus injection of (*S,S*)-[^18^F]FMeNER-D_2_. Venous blood samples were taken to measure the plasma concentration of duloxetine just before and after the second positron emission tomography scan.

**Results:**

Norepinephrine transporter occupancy by duloxetine was 29.7% at 20 mg, 30.5% at 40 mg, and 40.0% at 60 mg. The estimated dose of duloxetine inducing 50% norepinephrine transporter occupancy was 76.8 mg, and the estimated plasma drug concentration inducing 50% norepinephrine transporter occupancy was 58.0 ng/mL.

**Conclusions:**

Norepinephrine transporter occupancy by clinical doses of duloxetine was approximately 30% to 40% in human brain as estimated using positron emission tomography with (*S,S*)-[^18^F]FMeNER-D_2_.

**Significance Statement**

Norepinephrine transporter (NET) is one of the main targets in the treatment of depression. The radioligand (*S,S*)-[^18^F]FMeNER-D_2_ for imaging NET in the brain has been developed, and we have applied it in some clinical studies. Duloxetine is a serotonin and norepinephrine reuptake inhibitor that has been widely used for the treatment of depression. In this study, we investigated central NET occupancies using this ligand in 8 healthy subjects taking various doses of duloxetine. To our knowledge, this is the first study to measure NET occupancy by duloxetine in humans. The result showed approximately 30% to 40% NET occupancies in the brain by the administration of 20 to 60 mg of duloxetine. The findings will be of help in understanding the precise mechanisms of antidepressants.

## Introduction

The norepinephrine systems play important roles in various psychiatric disorders (Biederman and Spencer, 1999; Sara, 2009; Pietrzak et al., 2013). The norepinephrine transporter (NET), which modulates the amount of norepinephrine in the synaptic cleft, has been targeted for the treatment of neuropsychiatric disorders exemplified by major depressive disorder (MDD) (Nutt, 2006). Tricyclic antidepressants, some of which have a high affinity for NET, have been used to treat MDD (Bielski and Friedel, 1976). Clinical efficacies of serotonin and norepinephrine reuptake inhibitors such as milnacipran and duloxetine are reported to be equivalent or even greater than those of selective serotonin reuptake inhibitors in the therapy of MDD (Papakostas et al., 2007). However, finding optimal doses of drugs targeting NET for the treatment of MDD is still empirical, because information regarding optimal target occupancy is still limited (Nogami et al., 2013; Takano et al., 2014).

Development of a suitable positron emission tomography (PET) radioligand for NET, (*S,S*)-[^18^F]FMeNER-D_2_, has permitted the in vivo evaluation of NET density in the human brain (Schou et al., 2007; Takano et al., 2008), enabling the estimation of NET occupancy by antidepressants. So far, NET occupancy in the human brain by clinical doses of the antidepressants milnacipran and nortriptyline has been reported. NET occupancy by chronically administered 25 to 200 mg of milnacipran in patients with MDD was 25% to 50%, and more than 25% of NET occupancies might be effective for milnacipran treatment of MDD patients (Nogami et al., 2013). NET occupancies by a single low dose (10–75 mg) of nortriptyline in healthy human volunteers ranged from 16% to 41%, and the estimated plasma drug concentration inducing 50% NET occupancy (EC_50_) for this drug was reported to be 59.8 ng/mL (Sekine et al., 2010). In patients with MDD, NET occupancy by chronically administered moderate to high doses (75–200 mg) of nortriptyline was approximately 50% to 70%, and the EC_50_ value was 79.8 ng/mL (Takano et al., 2014). At least 50% of NET occupancy would be needed for the treatment of MDD with nortriptyline. More evidence for the relationship between pharmacological and pharmacodynamic properties of antidepressants acting on SERT and NET is required for the estimation and prediction of the therapeutic dosage of these drugs based on their occupancies of the targets.

Duloxetine is one of the first-choice antidepressants for MDD (Nemeroff et al., 2002). Our previous report demonstrated that a single dose of 40 mg of duloxetine, which is the minimum efficacy dose (Cipriani et al., 2012), induced nearly 80% occupancy of SERT (Takano et al., 2006), while the occupancies of NET by this or other dosages are yet to be determined. In this study, we investigated NET occupancy following various doses of duloxetine using PET with (*S,S*)-[^18^F]FMeNER-D_2_. This approach, along with previous PET assays, allows the clarification of relationships between occupancies of NET and SERT by selective and nonselective inhibitors providing antidepressant effects.

## Materials and Methods

### Participants

Eight healthy male subjects (25.5 ± 5.9 years, mean ± SD) participated in this study. All subjects were free of psychiatric, neurological, and somatic illness, and they had no history of drug abuse. Two subjects were current smokers. Studies were performed at the National Institute of Radiological Sciences, Chiba, Japan. All participants provided written informed consent before participating in the study, which was approved by the Radiation Drug Safety Committee and the Institutional Review Board of the National Institutes of Radiological Sciences.

### Acquisition of PET Images and Magnetic Resonance Images (MRI)

Two PET scans were performed for each subject. The first PET scan with (*S,S*)-[^18^F]FMeNER-D_2_ was performed with no medications. The second PET scan was performed 6 hours after single-dose oral administration of duloxetine. These 2 scans were performed at the same time on different days, and the average interval between the 2 scans was 16.5 (11.5 SD) days. (*S,S*)-[^18^F]FMeNER-D_2_ was synthesized by fluoromethylation of nor-ethyl-reboxetine with [^18^F]bromofluoro-methane-D_2_ (Schou et al., 2004). An ECAT EXACT HR+ (CTI-Siemens) PET scanner system, which provides a 15.5-cm axial field of view, together with a head fixation device to minimize head motion, was used for the 2 scans of all subjects. A 10-minute transmission scan was acquired after the emission scan using a ^68^Ge-^68^Ga source for subsequent attenuation correction. Emission data were in the 3-dimensional mode acquired from 120 to 180 minutes (10 minutes × 6 frames) after an i.v. bolus injection of (*S,S*)-[^18^F]FMeNER-D_2_. Data were reconstructed by filtered back-projection using a Hanning filter (6.3 mm, full width at half maximum). Injected radioactivity averaged 184.0 (23.1 SD) MBq, and specific radioactivity averaged 704.3 (127.0 SD) GBq/μmol at the time of injection.

MRI was performed with a 3.0-Tesla MR scanner, MAGNETOM Verio (SIEMENS). Three-dimensional volumetric acquisition of a T1-weighted gradient echo sequence produced a gapless series of thin sagittal sections (TE, 1.95 milliseconds; TR, 2300 milliseconds; TI, 900 milliseconds; flip angle, 9 degrees; field of view, 250 mm; acquisition matrix, 256×256; slice thickness, 1 mm; voxel size, 1 mm×1 mm×1 mm). We visually confirmed that there were no obvious abnormalities, including structural abnormalities, space occupying regions, vascular changes, and so on.

### Administration and Measurement of Plasma Concentration of Duloxetine

Each subject was given a single oral dose of 20 mg (n=3), 40 mg (n=3), or 60 mg (n=2) of duloxetine 6 hours before the second PET scan was started, because the median time to maximum plasma concentration was 6 hours after duloxetine administration (Knadler et al., 2011). Venous blood samples were taken to measure the plasma concentration of duloxetine just before and after the second PET scan. Plasma concentrations of duloxetine were determined by gas chromatography-mass spectrometry with a lower limit of quantification of 0.1 ng/mL by Sumika Chemical Analysis Service, Ltd. The average of these 2 plasma concentrations from before and after the scan was used for the following analysis.

### Image Analysis

Firstly, T1-weighted MRIs were co-registered to the corresponding summated PET images using SPM8 (Wellcome Trust Centre for Neuroimaging). Secondly, the MRIs were spatially normalized to the Montreal Neurological Institute space (MNI 152 2-mm template) by diffeomorphic anatomical registration using the exponentiated lie algebra algorithm in SPM8. We then applied those transformation parameters to the corresponding dynamic PET images.

For the thalamus, the anatomical automatic labeling template was used to place regions of interest (Tzourio-Mazoyer et al., 2002). White matter, which represents the segmentation of MRI by SPM, was used as the reference region, because it is almost devoid of specific binding of (*S,S*)-[^18^F]FMeNER-D_2_ to NET (Gulyas et al., 2010). To estimate radioligand binding, we calculated the ratio of the area under the time activity curve (AUC) at 120 to 180 minutes between the thalamus and white matter minus 1, which is termed AUC ratio −1. AUC ratio −1 at 120 to 180 minutes matched well with the binding potential values estimated by a 2-tissue compartment model fit to 240-minute dynamic scan data using an arterial input function (Moriguchi et al., 2016). NET occupancy was calculated by the following equation:

$$\text{Occupancy}(\text{\%})=\frac{{\left(\text{AUC ratio}-1\right)}_{baseline}-{\left(\text{AUC ratio}-1\right)}_{drug}}{{\left(\text{AUC ratio}-1\right)}_{baseline}}\;,$$

where (AUC ratio -1) _baseline_ is (AUC ratio -1) in the drug-free state, and (AUC ratio -1) _drug_ is (AUC ratio -1) after administration of duloxetine.

The relationship between the dose or plasma concentration of duloxetine and NET occupancy is described by the following equations:

$$\text{Occupancy}\left(\text{\%}\right)=\frac{D}{D+{\text{ED}}_{50}}\;\times \;100,$$

$$\text{Occupancy}\left(\text{\%}\right)=\frac{C}{C+{\text{EC}}_{50}}\;\times \;100,$$

where *D* and *C* are the dose and plasma concentrations of duloxetine, respectively. ED_50_ and EC_50_ are the dose and plasma concentrations inducing 50% NET occupancy, respectively. The classical Hill equation was used to describe the relationship between drug plasma concentration or dose and NET occupancy. We used Graph Pad prism ver 6 to calculate ED_50_ and EC_50_.

## Results

**Figure 1.**
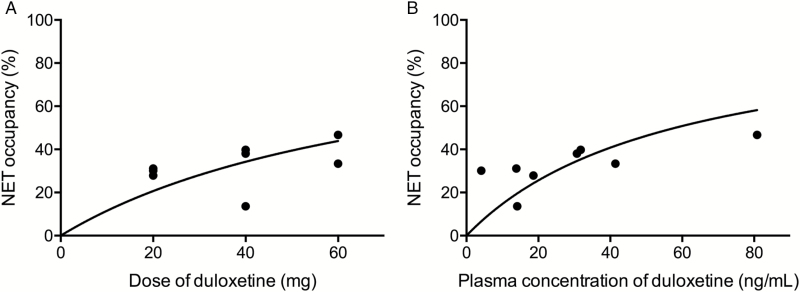
(A) Relationship between the occupancy of norepinephrine transporter (NET) and the doses of duloxetine. (B) Relationship between the occupancy of NET and the plasma concentration of duloxetine.

There was a significant correlation between the dose and plasma concentrations of duloxetine (Pearson’s r = 0.82, *P* = .012) (Table 1). The mean NET occupancies by duloxetine at 20, 40, and 60 mg were 29.7% (1.3 SD), 30.5% (11.9 SD), and 40.0% (6.7 SD), respectively. The relationship between NET occupancy and duloxetine dose is shown in Figure 1A. Estimated ED_50_ was 76.8 mg. The relationship between NET occupancy and the plasma concentration of duloxetine is shown in Figure 1B, and estimated EC_50_ was 58.0 ng/mL. A significant correlation between plasma concentration and duloxetine occupancy was found (r = 0.72, *P* = .044). The root mean square error of nonlinear fitting, which is the indicator of good fitness, was 11.8, and that of linear fitting was 7.3.

**Table 1.** Subject Demographics, Doses, and Plasma Concentrations of Duloxetine and Norepinephrine Transporter (NET) Occupancy in the Thalamus According to the Area-Under-The-Curve Ratio Method

Age (y)	Smoking	Duloxetine dose (mg)	Plasma duloxetine concentration (ng/mL)	NET occupancy (%)
23	No	20	4.1	30.2
41	No	20	18.7	27.9
24	No	20	13.9	31.1
23	Yes	40	30.7	38.0
22	No	40	31.8	39.8
23	Yes	40	14.1	13.6
24	No	60	41.4	33.4
24	No	60	80.8	46.7

## Discussion

In the present study, we measured NET occupancies by different doses of duloxetine in healthy subjects. The results showed that 20 to 60 mg of duloxetine induced 30% to 40% of NET occupancy.

NET occupancy by duloxetine was similar to that by milnacipran (Nogami et al., 2013) but slightly lower than that by nortriptyline (Takano et al., 2014), although all 3 drugs were given at clinically used doses. While nortriptyline is a NET-selective antidepressant, milnacipran and duloxetine are serotonin and norepinephrine reuptake inhibitors targeting both NET and SERT. Our previous PET study using [^11^C]DASB, a radioligand for SERT, indicated that SERT occupancies by duloxetine were 71.3% at 20 mg, 80.6% at 40 mg, and 81.8% at 60 mg (Takano et al., 2006). In light of those and the current findings, it is likely that the same dose of duloxetine induces a higher occupancy of SERT than that of NET. This could be explained by the lower affinity of duloxetine for NET (Ki = 7.5 nM) than for SERT (Ki = 0.8 nM) (Bymaster et al., 2001). Our EC_50_ value was 58.0 ng/mL, which was around 10 to 20 times that of 3.7 (Takano et al., 2006) or 2.62 ± 0.93 ng/mL (Abanades et al., 2011) reported by previous studies. The difference between SERT and NET occupancy could be explained by the difference in affinity.

Previous PET studies reported that SERT occupancy exceeding 80% was required for the effective treatment of MDD patients by SSRI (Meyer et al., 2001, 2004). Thus, the occupancy of SERT with duloxetine appears adequate for treatment of MDD and might complement the low NET occupancy, because MDD is a multi-pathology disease involving both NET and SERT (Gryglewski et al., 2014; Moriguchi et al., 2017). However, clinical dose of milnacipran showed low occupancy of both SERT and NET (Nogami et al., 2013). That indicated insufficient dose setting might be assumed in case of milnacipran.

The NET occupancy by 40 mg of duloxetine in one subject was lower than that in the other subjects taking the same dose. In this subject, the plasma concentration was also low, and this could be explained by the current smoking status of the subject, as smoking has been documented as reducing duloxetine plasma levels by inducing cytochrome P450 1A2 (Fric et al., 2008). However, the duloxetine plasma level of another smoker was not low, which might be explained by the difference in the amount of smoking. Unlike the dose-occupancy relationships, the relationship between the plasma level of duloxetine and NET occupancy by this drug is not affected by smoking.

In our data, linear fitting was better than nonlinear fitting. However, linear fitting is not theoretical, because occupancy over 100% is not realistic. Thus, we assumed that nonlinear fitting using the Hill equation is plausible. There could be 2 reasons why nonlinear fitting model is not the best. The reasons are that the number of data points are small and that we could not obtain NET occupancies close to the maximum (Emax), which are needed for a precise estimation of ED_50_ and EC_50_. In this study, achieving an occupancy near Emax was not possible, because the dose of duloxetine in the subjects could not exceed its clinical dose levels. Thus, further studies with larger sample sizes will increase the identification accuracy of NET occupancy by high doses of duloxetine. In a study with rats, the EC_50_ value estimated in rats treated with wide-range doses of duloxetine including a high dose inducing Emax was 59.0 ng/mL (Bourdet et al., 2012), a value similar to the EC_50_ determined in our study. These observations support the feasibility of the present methodology using in vivo data following a clinical dose of duloxetine and imply that EC_50_ of this drug for NET may be translatable between rats and humans, similar to its EC_50_ for SERT (Bourdet et al., 2012).

In this study, we used white matter as a reference region, whereas previous studies with (*S,S*)-[^18^F]FMeNER-D_2_ defined a reference in the caudate (Arakawa et al., 2008). We could obtain more robust quantitative measures with the white matter reference, as a large volume of white matter resulted in a low signal-to-noise ratio compared with the use of the caudate. Indeed, our previous assays showed that the white matter reference produced a smaller coefficient of variance of the (AUC -1) at 120 to 180 minutes in the midbrain and thalamus than the caudate reference (Moriguchi et al., 2016).

Our study has several limitations. First, our sample size was limited; thus, further studies with larger sample sizes will increase the accuracy of the identification of NET occupancy by variable doses of duloxetine. Second, we administered a single dose. The NET occupancy data obtained here may not be translated to subjects chronically taking duloxetine for an extended period. The discrepancy between single and multiple dosing was revealed in the relationship between plasma concentration and SERT occupancy of duloxetine in human (Abanades et al., 2011). Plasma concentration from 7 days of repeated administration of duloxetine would be assumed to be 16.2 ng/mL at 20 mg, 31.5 ng/mL at 40 mg, and 68.1 ng/mL at 60 mg at maximum serum concentration (Eli Lilly and Company, 2017). Based on our model, NET occupancy would be 21.8% with 20 mg, 35.2% with 40 mg, and 54.0% with 60 mg. These values are higher than with the single dose and would be adequate for treatment. Additional studies with participants being chronically administered duloxetine will be needed to more precisely estimate the NET occupancy of duloxetine.

In conclusion, our PET study with (*S,S*)-[^18^F]FMeNER-D_2_ has demonstrated that various single doses of duloxetine achieve NET occupancy ranging between 30% and 40%.

## Statement of Interest

None. Except for the governmental support mentioned in the statement of funding, there is no financial interest to disclose directly related to this work. Financial disclosures not directly related to the subject of this work are as follows: M.-R.Z., H.S., M.H., and T.S. hold a patent on compounds (JP 5422782/EP 12 884 742.3), and M.H. has an equity interest in Aprinoia Therapeutics, Inc. J.M. has received operating grant funds for other studies from Bristol-Myers Squibb, Eli Lilly and Company, GlaxoSmithKline, Lundbeck, and SK Life Sciences in the past 5 years. J.M. received operating grant funds for other studies from Janssen, Bristol-Myers Squibb, Eli Lilly and Company, and SK Life Sciences in the past 5 years. J.M has been a consultant to Mylan, Lundbeck, Takeda, Teva, and Trius in the past 5 years. J.M. is an inventor of 4 patented blood markers to predict brain inflammation and/or to diagnose affective disorders and a dietary supplement to reduce postpartum depressed mood. J.M. is arranging collaborations with nutraceutical companies for the dietary supplement. M.M. has received grants and/or speaker’s honoraria from Asahi Kasei Pharma, Astellas Pharmaceutical, Daiichi Sankyo, Dainippon-Sumitomo Pharma, Eisai, Eli Lilly, Fuji Film RI Pharma, Janssen Pharmaceutical, Kracie, Meiji-Seika Pharma, Mochida Pharmaceutical, MSD, Novartis Pharma, Ono Yakuhin, Otsuka Pharmaceutical, Pfizer, Shionogi, Takeda Yakuhin, Tanabe Mitsubishi Pharma, and Yoshitomi Yakuhin within the past 3 years. H.T. has received grants and/or speaker’s honoraria from Biogen Japan, Nihon Medi-Physics, Janssen Pharmaceutical, Tanabe Mitsubishi Pharma, and Mochida Pharmaceutical within the past 3 years. All authors declare no other conflicts of interest.
